# Regulation of ACVR1 and ID2 by cell-secreted exosomes during follicle maturation in the mare

**DOI:** 10.1186/1477-7827-12-44

**Published:** 2014-05-26

**Authors:** Juliano C da Silveira, Elaine M Carnevale, Quinton A Winger, Gerrit J Bouma

**Affiliations:** 1 Animal Reproduction and Biotechnology Laboratory, Department of Biomedical Sciences, Colorado State University, Fort Collins, CO, USA

**Keywords:** Follicular fluid, Ovarian follicle, Exosomes, miRNAs, Equine

## Abstract

**Background:**

Ovarian follicle growth and maturation requires extensive communication between follicular somatic cells and oocytes. Recently, intercellular cell communication was described involving cell-secreted vesicles called exosomes (50–150 nm), which contain miRNAs and protein, and have been identified in ovarian follicular fluid. The goal of this study was to identify a possible role of exosomes in follicle maturation.

**Methods:**

Follicle contents were collected from mares at mid-estrous (~35 mm, before induction of follicular maturation) and pre-ovulatory follicles (30–34 h after induction of follicular maturation). A real time PCR screen was conducted to reveal significant differences in the presence of exosomal miRNAs isolated from mid-estrous and pre-ovulatory follicles, and according to bioinformatics analysis these exosomal miRNAs are predicted to target members belonging to the TGFB superfamily, including *ACVR1* and *ID2*. Granulosa cells from pre-ovulatory follicles were cultured and treated with exosomes isolated from follicular fluid. Changes in mRNA and protein were measured by real time PCR and Western blot.

**Results:**

ACVR1 mRNA and protein was detected in granulosa cells at mid-estrous and pre-ovulatory stages, and real time PCR analysis revealed significantly lower levels of *ID2* (an ACVR1 target gene) in granulosa cells from pre-ovulatory follicles. Exposure to exosomes from follicular fluid of mid-estrous follicles decreased *ID2* levels in granulosa cells. Moreover, exosomes isolated from mid-estrous and pre-ovulatory follicles contain ACVR1 and miR-27b, miR-372, and miR-382 (predicted regulators of *ACVR1* and *ID2*) were capable of altering *ID2* levels in pre-ovulatory granulosa cells.

**Conclusions:**

These data indicate that exosomes isolated from follicular fluid can regulate members of the TGFB/BMP signaling pathway in granulosa cells, and possibly play a role in regulating follicle maturation.

## Background

Mammalian antral follicular development is the last step of folliculogenesis and culminates in ovulation or atresia [1]. This dynamic process requires extensive cross talk between follicular cells (theca, granulosa, cumulus and oocyte) [1-3], which is regulated by endocrine, paracrine and autocrine signaling. Recently, a new mechanism of intercellular communication mediated by cell-secreted vesicles was revealed [4]. Cell-secreted vesicles, called exosomes (~50-150 nm) and microvesicles (~100-1000 nm), carry bioactive material such as mRNAs, microRNAs (miRNAs), and proteins in different body fluids and deliver their contents to recipient cells [4]. Exosomes and microvesicles have been identified in ovarian follicular fluid of mares and cows, and these vesicles contain miRNAs and proteins [5,6]. MiRNAs, small (~22 nucleotide) non-coding RNAs, regulate gene expression by complementary base-pair interactions in the 3’ untranslated region of mRNA targets, leading to mRNA cleavage or translational repression [7,8]. Studies involving specific deletion of Dicer (necessary for mature miRNA synthesis) in ovaries demonstrate miRNAs are necessary for adult ovarian function and fertility [9]. In addition, miRNA-21 is involved in regulation of granulosa cell apoptosis and corpus luteum formation in mice [10], and gonadotropins regulate miRNA expression and consequently control estradiol production in sheep [11]. Finally, studies in mice reveal that transforming growth factor B (TGFB) induces miR-224 and miR-383 expression, which target *Smad4* and *Rbms1* thereby regulating estradiol production in response to gonadotropin stimulation [12,13]. These studies clearly indicate that miRNAs are important regulators of ovarian function by controlling various aspects of follicular growth and development, and also demonstrate a role for the TGFB signaling pathway in regulating miRNA transcription, as well as miRNAs controlling TGFB family members expression and function during folliculogenesis [12].

The TGFB/BMP signaling family is necessary for follicle development and oocyte competence in mammals. Different studies have demonstrated the role of specific family members in theca cells, granulosa cells, cumulus cells and oocytes [2,14]. Activins/Inhibins, BMPs, and GDFs are responsible for modulating the effects of both FSH and LH during all stages of follicle development. Therefore, understanding the mechanisms involved in regulating these signaling pathways is important to provide insight into the process of follicle growth and development and oocyte maturation.

Follicle development in the mare has been well described [15], and is characterized by follicular waves. Emergence of a follicular wave in the mare is defined by the presence of follicles between 6 and 13 mm in diameter, and deviation occurs in this growing cohort of follicles when an (immature) follicle obtains a diameter of ~22 mm while growth of subordinate follicles becomes static. The follicle reaches ~35-45 mm before ovulation is induced with a prolonged, periovulatory LH surge.

The overall goal of this study was to obtain a better understanding of the role of exosomes in follicle development and growth, and test the hypothesis that exosomes isolated from follicular fluid modulate TGFB/BMP signaling in granulosa cells. A miRNA profiling screen on exosomes isolated from mid-estrous and pre-ovulatory follicles identified miRNAs that are predicted to regulate the TGFB/BMP signaling members. In this study, we examined ACVR1 and ID2, two predicted targets of exosomal miRNAs, in granulosa cells and exosomes in follicular fluid of mid-estrous and pre-ovulatory ovarian follicles. In addition, we determined if exosomes isolated from follicular fluid of mid-estrous and pre-ovulatory follicles are capable of altering gene expression in pre-ovulatory granulosa cells.

## Methods

### Collection of ovarian follicular cells and fluid

Follicular fluid (10 ml) and granulosa cells were aspirated from dominant follicles (~35 mm before induction of follicular maturation - mid-estrus), and (30–34 h after induction of follicular maturation - pre-ovulatory) from young (3–12 yr) estrous mares (*Equus Caballus*) that were part of the clinical program at the Equine Reproduction Laboratory, CSU. All procedures were done in accordance with the Colorado State University Institutional Animal Care and Use Committee. Mares were housed on dry lots with water and hay. Reproductive tracts of the mares were examined using transrectal ultrasound. A synthetic prostaglandin analog cloprostenol sodium (250 mg of Estrumate® Merck, NJ, USA) was administered during two consecutive days beginning on day 5 or 7 following ovulation or aspiration. Mid-estrus dominant follicles were determined based on follicle growth and diameter (ultrasound), relaxed cervical tone and endometrial edema. For pre-ovulatory follicle collections, follicular maturation was induced by administration of hCG and/or deslorelin (2500 IU and 1.5 mg, respectively, iv), and follicular contents were collected 32 h later by transvaginal, ultrasound-guided follicular aspirations using a 12-GA needle [16,17]. A sample of follicular fluid was collected and centrifuged at 300 × g for 10 min, 2000 × g for 10 min, 10000 × g for 30 min and later stored at -80°C until processed for exosome isolation. The follicle antrum was rinsed to recover granulosa cells. Red blood cells were removed by rinsing granulosa cells (3×) in PBS containing 0.02% polyvinyl alcohol (PVA). Approximately half of the sample was snap frozen and used for total RNA and protein isolation, whereas the rest of the granulosa cells were pipetted repeatedly to separate cells and placed in DMEM/F-12, (Invitrogen™ #11320-033, Carlsbad, CA, USA) with no addition of fetal bovine serum (FBS). Cells were cultured for 24 h and used in granulosa cell culture and exosome treatment experiments (see below).

### Isolation of exosomes from follicular fluid

Exosomes were isolated from ovarian follicular fluid starting with the three first steps of differential centrifugation as described [18], followed by Exoquick™ (SBI System Biosciences, Inc, Mountain View, CA, USA) precipitation, a polymer-based reagent that precipitate exosomes. Briefly, following centrifugation, 400 μl of follicular fluid supernatant was added to 100 μl of Exoquick. This preparation was incubated overnight at 4°C and centrifuged at 1500 × g for 30 min to obtain an exosome pellet. Exosome pellets were resuspended in 250 μl of PBS (pH 7.4) and used for miRNAs real time PCR analysis (n = 6 mares at mid-estrus, and n = 6 mares at pre-ovulation).

### Granulosa cell culture and exosome treatment

Granulosa cells from pre-ovulatory follicles were placed in 24-well dishes (Nunc, Inc. #142475, Waltham, MA, USA) at 37°C and density of 5×10^6^ cells per well in 2 ml of DMEM/F-12 medium without FCS. Granulosa cells attached to the dishes and were confluent. Cells were cultured for 24 h and exposed to exosomes for 24 h (totalizing 48 h in culture). These culture conditions were modified based on Davidson, et al., 2002 [19] Medium was replaced after the first day to remove any dead cells, and treatment started on the second day. Exosomes isolated from 800 μl of follicular fluid were resuspended in 250 μl of media. Treatment was composed of 1.75 ml of medium and 250 μl of resuspended exosomes. Control was composed of 2 ml of medium without the addition of exosomes. Granulosa cells from pre-ovulatory follicles (n = 4 mares) were placed in culture independently and treated with exosomes isolated from follicular fluid from pre-ovulatory (n = 4 mares) or mid-estrus (n = 4 mares) follicles, or not treated with isolated exosomes. Cells and exosomes were collected and used for total RNA and protein isolation.

### Western blot analysis

Granulosa cell and exosomal proteins were isolated using TRI Reagent®BD (Molecular Research, Inc.) according the manufacturer’s instructions, and exosomal proteins were resuspended in 8 M urea. Protein concentrations were determined using the Bradford assay. A total of 30 μg of protein was loaded and resolved in 12% SDS-PAGE polyacrylamide gels (Bio-Rad, Hercules, CA, USA). Protein samples were run at 30 mA for 45 min and transferred to nitrocellulose membranes (Biotrace NT, Pall life Sciences, Pensacolla, FL, USA) for 1 h at 100 V. Membranes were incubated in blocking buffer (5% non-fat dried milk in TBST) for 2 h at room temperature, and the presence of ACVR1 was assessed by exposing membranes to a goat polyclonal antibody raised against a peptide mapping to the N-terminus of ACVR1 of human origin (0.4 μg/ml, sc-5671, Santa Cruz Biotechnology Inc., Santa Cruz, CA, USA) overnight at 4˚C. In addition, membranes were exposed to ACTB mouse monoclonal antibody raised against ACTB of avian origin (0.1 μg/ml, sc-47778, Santa Cruz Biotechnology Inc., Santa Cruz, CA, USA), as a reference protein. Membranes were washed three times in 1X TBST for 5 min, and incubated for 1 h at room temperature with a horseradish peroxidase conjugated anti-goat secondary antibody (0.2 μg/ml, sc-2020, Santa Cruz Biotechnology Inc., Santa Cruz, CA, USA) or horseradish peroxidase conjugated anti-mouse secondary antibody (1 μg/ml, ab-6789, Santa Cruz Biotechnology Inc., Santa Cruz, CA, USA), respectively (Table 1). Membranes were washed three times in 1X TBST for 5 min and incubated for 5 min in ECL Plus Prime Western Blotting Detection System solution (Amersham™, Buckinghamshire, UK) for color development, and image and band analyses were performed using ChemiDoc MP Image System (Bio-Rad, Hercules, CA, USA).

**Table 1:** Antibodies utilized for Western blot analysis

**Protein target**	**Antibody**	**Catalog number**	**Accession number**	**Species raised**	**Dilution used**
ACVR1	Antibody raised against a peptide mapping to the N-terminus of ACVR1 of human origin	sc-5671	Q04771	Goat polyclonal	0.4 μg/ml
	Horseradish peroxidase conjugated anti-goat secondary antibody	sc-2020			0.2 μg/ml
ACTB	Antibody raised against ACTB of avian origin	sc-47778	P60709	Mouse monoclonal	0.1 μg/ml
	Horseradish peroxidase conjugated anti-mouse secondary antibody	ab-6789			1 μg/ml

### mRNA and miRNA isolation and reverse transcription

Total RNA including miRNA was isolated from exosome preparations using TRI Reagent ®BD (Molecular Research, Inc., Cincinnati, OH, USA), according to the manufacturer’s instructions. RNA concentration and purity were determined using the NanoDrop ND-1000 spectrophotometer. Samples were stored at -80°C until further use.

Quantifiable, reverse transcribed mRNA was generated using qScript™ cDNA Synthesis Kit (Quanta Biosciences Cat#95047, Gaithersburg, MD, USA), which contains both random and oligo (dT) primers, according to the manufacture’s instructions. Briefly, the reverse transcription reaction was carried out using approximately 30 ng of total RNA for each selected gene. RNA was incubated with 5× qScript Reaction Mix, qScript Reverse Transcriptase, and DNase-free water at 22°C for 5 min, at 42°C for 30 min, and at 85°C for 5 min.

Quantifiable, reverse transcribed miRNAs were generated using the miScript PCR System (Qiagen® #218193, Venlo, Limburg, Netherlands) according to the manufacturer’s instructions. Briefly, the reverse transcription reaction was carried out with approximately 100 ng of total RNA for 384 miRNAs. Total RNA, including the small RNA fraction, was incubated with 5X miScript HiFlex Buffer, 10X miScript Nucleic Mix, RNase-free water, and miScript Reverse Transcriptase at 37°C for 60 min followed by 5 min at 95°C.

### Real-time PCR expression analysis of mRNAs and miRNAs

Relative levels of mRNAs were examined in granulosa cells recovered from ovarian follicles of young mares at mid-estrus (n = 6 mares) and pre-ovulation (n = 6 mares). In addition, mRNAs levels were examined in granulosa cells from pre-ovulatory follicles exposed in culture for 24 h to exosomes isolated from follicular fluid collected at mid-estrus or pre-ovulation. Gene specific primers were designed using Primer3 - BioTools software and are presented on Table 2. Each analysis was performed in 10 μl reactions containing 2X SYBR Green I master mix (Roche Applied Sciences, Indianapolis, IN, USA), 0.5 μM of forward and reverse primers (primers were designed against equine gene sequences, and are provided in Table 2), and 1 μl cDNA. The PCR cycle conditions were as followed: 95°C for 5 min, 45 cycles of 95°C for 10 sec, 60°C for 15 sec, and 72°C for 15 sec, followed by melt curve analysis to confirm amplification of single cDNA products. To identify differences in mRNA levels in granulosa cells before treatment or after treatment with exosomes from mid-estrus (n = 4 mares) or pre-ovulatory (n = 4 mares) follicles, raw Ct values were normalized to the geometrical mean of two internal controls (*ACTB*, *GAPDH*) previously used in different reproductive tissues [20,21], and statistical differences were assessed using a Student’s t-test. Geometric mean of the two selected internal controls was 27 and 18 in granulosa cells for the in vivo and in vitro experiments, respectively.

**Table 2:** Primers used for real-time PCR analysis and normalized Ct values in granulosa cells

**Gene**	**Primer forward: 5′-3’**	**Primer reverse: 5′-3**	**Accession number**	**Average (Δ) Ct ± SEM**	**Annealing temperature °C**	**Fragment size (bp)**
*ACVR1*	CCTCTCCTGTGGGAATGAGG	CTGGAAGCAGCCTTTCTGGT	XM_001491549.3	8.95 ± 0.32	60	100
*ACVR2B*	ATGTACCGGCATCGAAAACC	CGAGCCTTGATCTCCAACAG	XM_001488736.4	13.99 ± 0.39	60	119
*TGFBR2*	GACCCCAAGCTCACCTACCA	TGCACTCATCAGAGCTACAGGA	XM_005600872.1	0.8 ± 0.43	60	124
*CDKN2B*	CCGAGCTGCTACTGCTCCAC	CACCAGCGTGTCCAGGAAG	XM_001496235.3	13.93 ± 0.70	60	107
*COL1A2*	AGGTTTCCAAGGACCTGCTG	GGTTTTCCAGGGTGACCATC	XM_005609220.1	16.21 ± 0.75	60	117
*COL3A1*	GTCCCAACCCAGAGATTCCA	CGCTACTTTCATTTCCTTTCAGG	XM_001917620.3	2.05 ± 0.57	60	102
*USF2*	GGATACCACGGCTGTGTCAG	ATCGTCCTCTGCGTTCCTGT	XM_005596152.1	4.23 ± 0.70	60	114
*IL6*	GGCAGAAAAAGACGGATGCT	CACCCTTGAACTCGTTCTGGA	XM_005609172.1	13.03 ± 0.38	60	122
*ITGB7*	TGCCGAAGGATACCCTGTAGA	CTGCAGCTTCTCCAGCAAGG	XM_005611222.1	14.58 ± 0.82	60	112
*TGFB1*	CTCAGTGCCCACTGCTCCT	CATCAATGGTGGCCAGATCA	XM_005596086.1	2.68 ± 0.27	60	100
*ID1*	ACATGAACGGCTGCTACTCG	TCCAACTCCAGGTCCCAGAT	XM_005604549.1	10.02 ± 0.51	60	124
*ID2*	CATCCCCCAGAACAAGAAGG	TGGTGATGCAGGCTGACAAT	XM_001503611.3	2.37 ± 0.18	60	180
*ACTB*	CGACATCCGTAAGGACCTGT	CAGGGCTGTGATCTCCTTCT	NM_001081838.1|		60	99
*GAPDH*	AGAAGGAGAAAGGCCCTCAG	GGAAACTGTGGAGGTCAGGA	NM_001163856.1		60	87

The relative levels of 384 mature miRNAs were examined in exosome preparations isolated from follicular fluid of different mares at mid-estrus (n = 6 mares) and pre-ovulatory (n = 6 mares), using Human miRNome Profiler plates (SBI) with miRNA sequences conserved between horses and humans. Based on the initial PCR screen, miRNAs were considered for second analysis if present in at least 3 out of six samples and significantly different between groups. Selected miRNAs were analyzed using DIANA TOOLS [22] as a group since they were contained within exosomes. Based on the initial screen, levels of mature miRNAs predicted to target members of the TGFB superfamily were examined in granulosa cells and exosomes isolated from follicular fluid, as well as cells in culture after treatment with exosomes from mid-estrus follicles (n = 4 mares) and pre-ovulatory follicles (n = 4 mares). Each analysis was performed in 6 μl reactions containing 2X SYBR Green I master mix (Roche Applied Sciences, Indianapolis, IN, USA), 10 μM Universal reverse primer (Qiagen, Venlo, Limburg, Netherlands) and miRNA specific forward primer, and 0.03 μl cDNA. Real-time PCR was conducted using the LightCycler480 PCR system (Roche Applied Sciences, Indianapolis, IN, USA) in 384-well plates. The PCR cycle conditions were as followed: 95°C for 5 min, 45 cycles of 95°C for 10 sec, 55°C for 15 sec, and 72°C for 15 sec followed by a melt curve analysis to confirm amplification of single cDNA products. To identify differences in the presence of exosomal miRNAs isolated from follicular fluid or miRNAs in granulosa cells, raw Ct values were normalized to miR-99b; invariable present in all samples (mid-estrous Ct = 19.5 ± 0.6, pre-ovulatory Ct = 19.7 ± 0.5).

### Statistical analysis

Data presented a normal distribution and statistical differences were assessed using an unpaired Student’s t-test. Normalized data were compared between follicular stage groups, using the average of at least three samples per group and data were plotted as 1/ΔCt.

## Results

### Presence of ACVR1 and ID2 during antral follicle development

**Figure 1:**
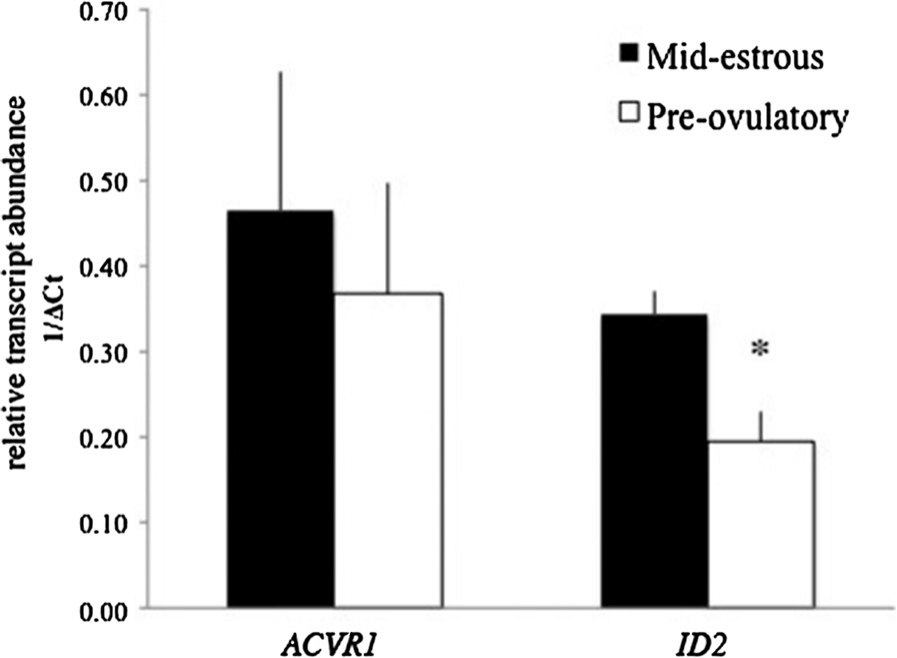
**Relative level of ACVR1 and ID2 in equine granulosa cells collected from mid-estrous and pre-ovulatory follicles.** Values on y-axis indicate 1/normalized (Δ) Ct values relative to geometric mean of *ACTB* and *GAPDH*. * = P < 0.05 between follicular stage.

**Figure 2:**
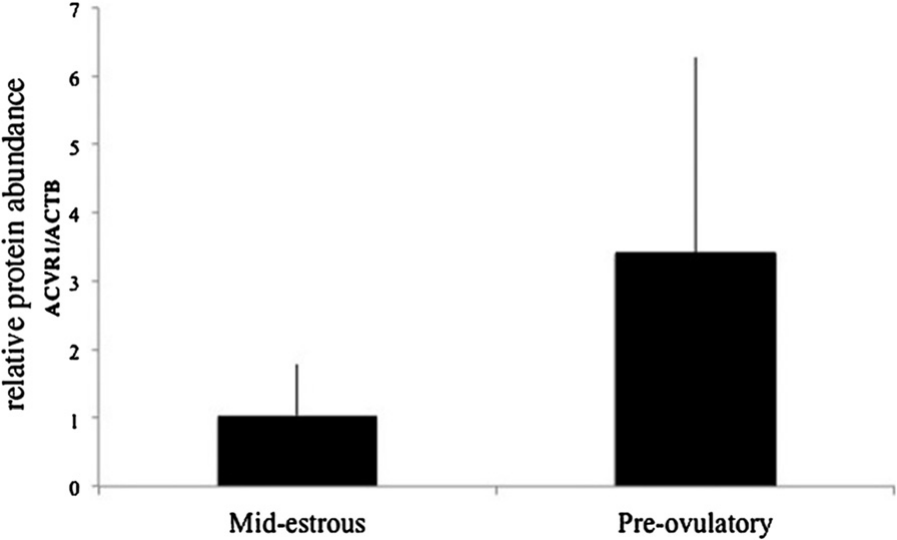
**ACVR1 protein levels in granulosa cells isolated from mid-estrous and pre-ovulatory follicles.** Values on y-axis indicate normalized values relative to ACTB.

An exosomal miRNA PCR screen was conducted from mature follicles and bioinformatics analyses identified miRNAs predicted to target and regulate genes of the TGFB/BMP signaling family. Twelve TGFβ family members, including receptors (*ACVR1*, *ACVR2B*, *TGFR2*), TGFB/Activin responsive genes (*COL1A2*, *COL3A1*, *CDKN2B*, *USF2*, *IL6*, *ITGB7*, *TGFB1*), and BMP-responsive genes (*ID1*, *ID2*), were selected for real time PCR analysis to examine their relative level in granulosa cells. These transcripts were detected (Ct < 37) in granulosa cells before treatment with exosomes (data not shown). Relative levels of *ID2* were ~2 fold lower (P < 0.02) in granulosa cells from pre-ovulatory compared to mid-estrous follicles (Figure 1). The BMP receptor *ACVR1*, involved in regulating ID2 expression, was present at similar levels in granulosa cells collected at mid-estrous compared to pre-ovulatory samples (Figure 1), and ACVR1 protein was present in granulosa cells from mid-estrous and pre-ovulatory follicles (Figure 2).

### Relative levels of miRNAs predicted to target ACVR1 and ID2 in granulosa cells and exosomes

**Figure 3:**
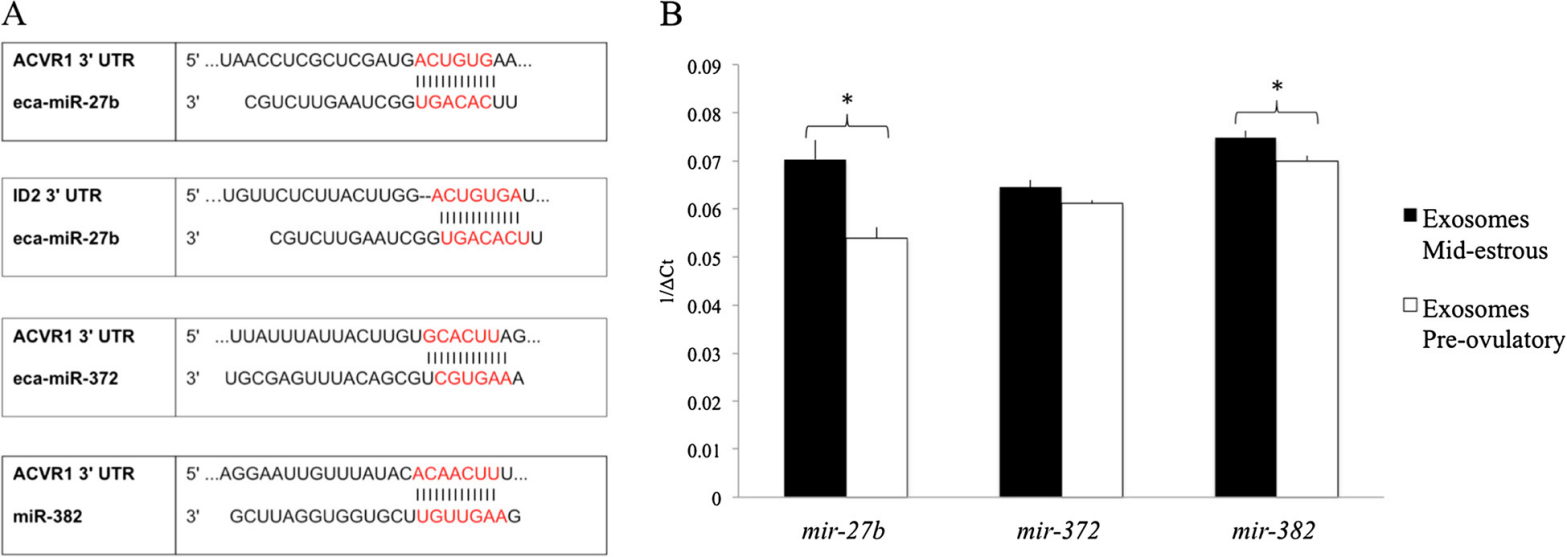
**Exosomal miRNAs predicted to regulate ACVR1 and ID2 transcripts. (A)** MiR-27b predicted binding sites in the 3’UTR of equine *ACVR1* and *ID2*, and miR-372 and miR-382 predicted binding sites in the 3’UTR of equine *ACVR1*. **(B)** Relative levels of miR-27b, miR-372, and miR-382 in exosomes isolated from follicular fluid of mid-estrous and pre-ovulatory follicles that are predicted to target *ACVR1* and/or *ID2*. Values on y-axis indicate 1/normalized (Δ) Ct values relative to geometric mean of *ACTB* and *GAPDH* or miR-99b and protein ratio. * = P < 0.05 between follicular stage.

Following an initial 384 miRNA PCR screen, we focused on miRNAs predicted to target *ACVR1* (e.g., miR-27b, miR-372, miR-382) and *ID2* (miR-27b) in this study (Figure 3A). Interestingly, miR-382 only was detected in granulosa cells from mid-estrus follicles and not in granulosa cells from pre-ovulatory follicles, and miRNA-27b was detected in granulosa cells and cumulus cells [5]. MiR-27b, miR-372, and miR-382, predicted to target *ACVR1* and *ID2,* were detected by real time PCR in exosomes isolated from follicular fluid collected from mid-estrous and pre-ovulatory follicles (Figure 3B). Relative levels of miR-27b and miR-382 were higher (P < 0.05) in exosomes collected from follicular fluid of mid-estrous compared to pre-ovulatory follicles.

### Granulosa cell treatment with exosomes isolated from follicular fluid from mid-estrous and pre-ovulatory follicles

**Figure 4:**
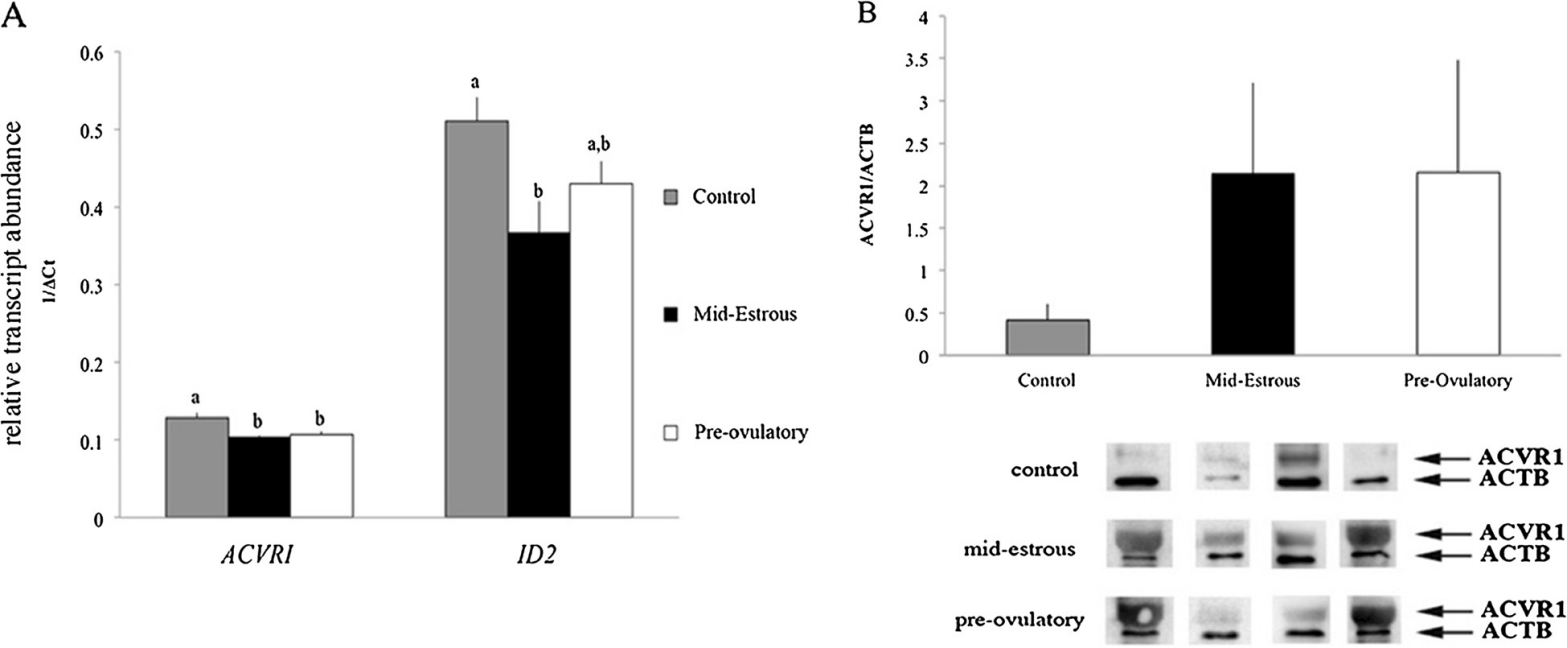
**Levels of ACVR1 mRNA and protein and ID2 mRNA in granulosa cells following exosomal treatment. (A)** Relative levels of *ACVR1* and *ID2* in granulosa cells following treatment with exosomes isolated from follicular fluid of mid-estrous and pre-ovulatory follicles. Values on y-axis indicate 1/normalized (Δ) Ct values and relative to geometric mean of *ACTB* and *GAPDH*. Different letters indicate P < 0.05 compared to control (no exosome treatment). **(B)** ACVR1 protein level in pre-ovulatory granulosa cells following treatments with follicular fluid exosomes. Replicates are composed of granulosa cells from pre-ovulatory follicles (n = 4) and exosomes isolated from follicular fluid of pre-ovulatory (n = 4) and mid-estrous follicles (n = 4). Bottom panels indicates the Western blot images with (upper band) ACVR1 and (lower band) ACTB.

To determine if exosomes from different follicular stages can affect relative level of TGFB/BMP family members in granulosa cells, exosomes isolated from follicular fluid of mid-estrous and pre-ovulatory follicles were added to pre-ovulatory granulosa cells in culture. Following a 24 h incubation period, mRNA levels of TGFB members were examined by real time PCR analysis (Additional file 1: Figure S1). *ACVR1* levels in granulosa cells were lower (P < 0.05) following treatment with exosomes isolated from follicular fluid of mid-estrous and pre-ovulatory follicles compared to control granulosa cells (Figure 4A). Similarly, relative levels of *ID2* also were altered following exosomes treatment; *ID2* levels decreased (P < 0.05) following exosome treatment from mid-estrous follicles compared to control granulosa cells (Figure 4A).

To complement changes observed in mRNA levels, relative protein levels were determined using Western blot analysis. Initial efforts focused on ACVR1, and Western blot analysis detected a band of ~ 50 kDa band. Treatment of granulosa cells with exosomes collected from follicular fluid of mid-estrous and pre-ovulatory follicles tended to increase ACVR1 protein (P < 0.07) compared to control granulosa cells; however, Western blot results were variable (Figure 4B).

**Figure 5:**
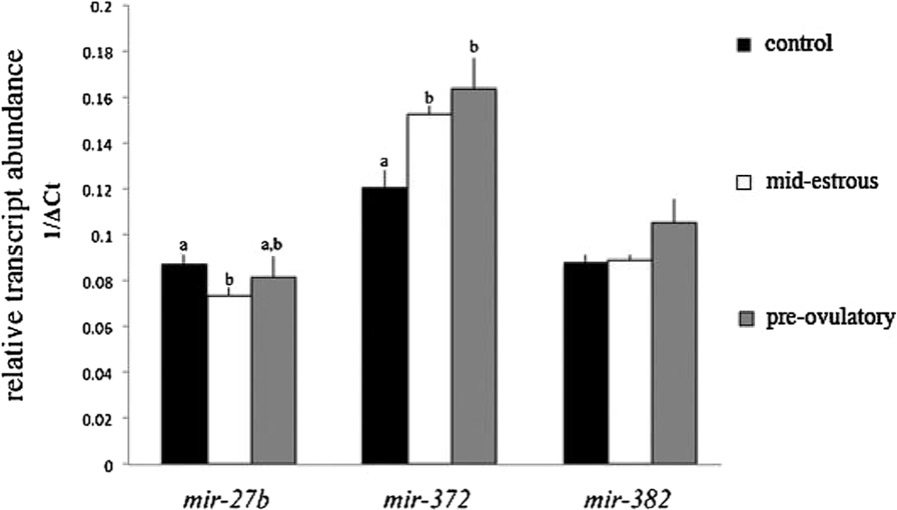
**MiRNA-27b, miR-372, and miR-382 levels in pre-ovulatory granulosa cells following exosome treatment.** Values on y-axis indicate 1/normalized (Δ) Ct values relative to miR-99b. Different letters indicate P < 0.05.

Relative levels of miRNAs predicted to regulate TGFB family members, including *ACVR1* and *ID2,* were examined in granulosa cells following treatment with exosomes isolated from follicular fluid of mid-estrous and pre-ovulatory follicles (Figure 5). MiR-372 levels increased (P < 0.05) in cultured granulosa cells following treatment with exosomes isolated from mid-estrous and pre-ovulatory follicles compared to control granulosa cells (Figure 5). In addition, miRNA-27b was lowered (P < 0.05) in cultured granulosa cells after treatment with exosomes isolated from mid-estrus follicles compared to cultured granulosa cells not exposed to exosomes (Figure 5).

### Presence of ACVR1 in exosomes

**Figure 6:**
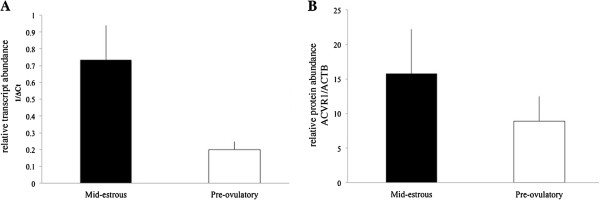
**ACVR1 mRNA and protein level in exosomes. (A)** Relative levels of ACVR1 in exosomes isolated from mid-estrous and pre-ovulatory follicles. Values on y-axis indicate 1/normalized (Δ) Ct values relative to geometric mean of *ACTB* and *GAPDH*. **(B)** ACVR1 protein level in exosomes isolated from mid-estrous and pre-ovulatory follicles.

Presence of ACVR1 mRNA and protein in exosomes was assessed using real time PCR and Western blot analysis. *ACVR1* levels were ~ 3.7 fold lower (P = 0.09) in exosomes collected from follicular fluid of pre-ovulatory follicles (Figure 6A). Similarly, ACVR1 protein was present in exosomes isolated from mid-estrous and pre-ovulatory follicles, and although average protein levels appeared approximately twice as high in exosomes isolated from follicular fluid at mid-estrus, this was not significant (P = 0.4; Figure 6B).

## Discussion

The goal of this study was to demonstrate that exosomes isolated from follicular fluid of equine ovarian follicles at mid-estrous and pre-ovulatory stages could regulate gene expression in granulosa cells during follicular maturation. Initially this was based on bioinformatics analysis, which revealed that exosomal miRNAs are predicted to target and regulate 44 genes of the TGFB/BMP signaling family. Of the initial 12 genes profiled in equine granulosa cells, we focused on *ACVR1* and *ID2*.

ACVR1 (also known as ALK2) is a receptor involved in regulating *ID2* expression and the BMP response pathway, which in turn is known for its importance during follicular development [2,14]. During bovine follicle development, both FSH and estradiol regulate *ACVR1*, and BMP7 signaling through ACVR2A/ACVR1 in granulosa cells is thought to play a role in follicle growth [23]. Furthermore, *ACVR1* levels in granulosa cells increase with follicle size [23,24]. Similarly, *ID2* levels increase during follicle development in hens, and are highest in fully differentiated granulosa cells and cumulus-oocyte-complexes in pigs. It has been proposed that ID2 is necessary for increasing LH receptor levels [25,26]. Interestingly, in addition to BMP/ACVR1 signaling, CCAAT enhancer binding protein (C/EBP)B also regulates *Id2* expression [27], and C/EBPB knockout female mice are sterile and exhibit a failure of granulosa cell differentiation in periovulatory follicles [28]. In this study we detected ACVR1 mRNA and protein in equine granulosa cells collected from mid-estrus and pre-ovulatory follicles. Although *ACVR1* levels were not different between mid-estrous and pre-ovulatory granulosa cells, average ACVR1 protein levels appeared ~3 fold higher (not significantly) in pre-ovulatory granulosa cells. ACVR1 increases with follicular size; however, no significant difference was observed between immature and mature follicles in cattle [24].

Based on previous findings that miRNA-148a regulates ACVR1, and consequently down-regulate SMADs and *ID2 *[29], we determined the relative level of miRNAs predicted to target *ACVR1* and *ID2* in both granulosa cells and follicular fluid exosomes. We were unable to detect miR-148a in isolated exosomes from equine ovarian follicular fluid, but did identify other exosomal miRNAs predicted to target *ACVR1* and *ID2*. MiRNA-27b (predicted regulator of *ACVR1* and *ID2*) was not detected in granulosa cells consistently, however was detected at significantly higher levels in exosomes isolated from mid-estrous follicles. The source of exosomal miR-27b in follicular fluid is unclear. Analyzing miRNA cDNA libraries obtained from sheep ovarian follicles [30] revealed that medium size follicles have increased abundance of miR-27b compared to pre-ovulatory follicles, although the definite follicular cell type that expresses this miRNA is not known [11]. Interestingly, miR-27b was present at higher levels in cumulus cells from pre-ovulatory follicles of young compared to old mares [5].

MiR-372 and miR-382 (predicted regulators of *ACVR1*) levels were elevated in granulosa cells from mid-estrous follicles compared to pre-ovulatory follicles (miR-382 was not detected in granulosa cells from pre-ovulatory follicles). This expression pattern was recapitulated in exosomes collected from follicular fluid. In addition to *ACVR1*, miR-372 is involved in regulation of cell cycle inhibitors p21, p27 and p53 [31], that affect cell proliferation and differentiation. Interestingly, miR-382 has been identified in the ovarian cortex of bovine, and not in the cumulus cells or corpus luteum, suggesting this miRNA originates from theca or stromal cells [32].

To further investigate a possible function for exosomes on granulosa cells, we performed an *in vitro* experiment using pre-ovulatory granulosa cells and treatment with or without exosomes isolated from follicular fluid of mid-estrous or pre-ovulatory follicles. Initially, we evaluated the level of *ACVR1* and demonstrate that levels were significantly decreased in pre-ovulatory granulosa cells following exposure to exosomes from either mid-estrous or pre-ovulatory follicles. Combined with the observation that follicular fluid exosomes contain miR-372 and miR-382, and levels increase in granulosa cells following exosome treatment, it is possible these exosomal miRNAs regulate *ACVR1* levels in pre-ovulatory granulosa cells. Moreover, miR-27b also is predicted to target *ID2*, which was down-regulated in pre-ovulatory granulosa cells following exosomes treatment. Analysis of ACVR1 protein level in pre-ovulatory granulosa cells indicates ACVR1 levels are ~ 3 fold higher compared to mid-estrous granulosa cells. This could be explained if exosomes contain ACVR1 themselves and is transferred to granulosa cells. Previously, *ACVR1* was identified in exosomes originating from MC/9 murine cells and SW480 human colorectal carcinoma cells [33,34]. We observed exosomes from mid-estrous follicles had higher levels of ACVR1 mRNA (~3.7 fold) and protein (~1.8 fold) compared to exosomes from pre-ovulatory follicles. However, the relative level of ACVR1 protein was quite variable in exosomes, which could be due to heterogeneous population of follicular fluid derived exosomes, and it is unclear if granulosa cells, cumulus cells, or both release exosomes containing ACVR1. Overall these data indicate that exosomes isolated from follicular fluid of mid-estrous follicles possibly transfer ACVR1 mRNA and/or protein.

## Conclusions

In the current study we identified a role of exosomes in regulating TGFB/BMP signaling in follicular cells. In vitro experiments using exosome treatments to granulosa cells revealed altered levels of a number of TGFB/BMP family members, and indicate a role for exosomes in regulating ACVR1 signaling and ID2 function in granulosa cells during follicle maturation possibly through direct delivery of ACVR1 present in exosomes. Future experiments will focus on identifying exosomes as delivery vehicles of TGFB/BMP family members.

## Competing interests

The authors declare that there is no conflict of interest that could be perceived as prejudicing the impartiality of the research reported.

## Authors’ contributions

JS conceived the work, performed sample collection, experimental design, experiments, data analysis, co-wrote the manuscript; EC managed the horses, follicle selections and experimental design; QW contributed reagents and assisted with the manuscript; GJB conceived and supervised the work, co-wrote the manuscript. All authors read, corrected and approved the final manuscript.

## Supplementary Material

Additional file 1: Figure S1 Relative level of selected TGFB/BMP signaling members in equine granulosa cells following treatment with exosomes isolated from follicular fluid of mid-estrous and pre-ovulatory follicles. Data is normalized data using the geometric mean of ACTB and GAPDH, and presented relative to 1. Different letters indicate P < 0.05. Click here for file
