# Supplementary figures and images for: Regulation of ACVR1 and ID2 by cell-secreted exosomes during follicle maturation in the mare
Source: Reprod Biol Endocrinol. 2014 May 26;12:44. doi: 10.1186/1477-7827-12-44 (PMC4045866; doi:10.1186/1477-7827-12-44)

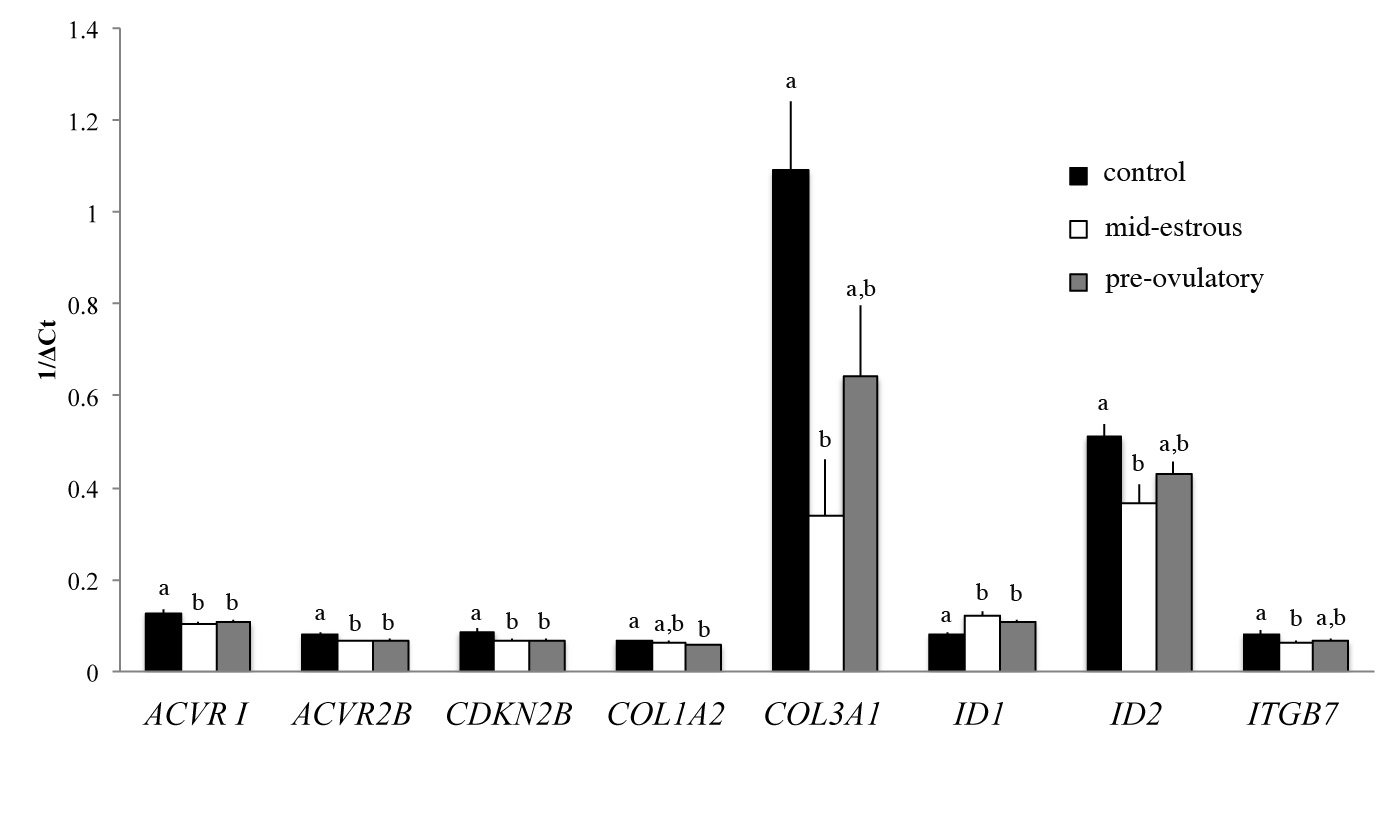

Supplement: Additional file 1: Figure S1 — Relative level of selected TGFB/BMP signaling members in equine granulosa cells following treatment with exosomes isolated from follicular fluid of mid-estrous and pre-ovulatory follicles. Data is normalized data using the geometric mean of ACTB and GAPDH, and presented relative to 1. Different letters indicate P < 0.05. [file 1477-7827-12-44-S1.jpeg]
